# Co-sequencing and novel delayed anti-correlation identify function for pancreatic enriched microRNA biomarkers in a rat model of acute pancreatic injury

**DOI:** 10.1186/s12864-018-4657-2

**Published:** 2018-04-27

**Authors:** Zhihua Li, Rodney Rouse

**Affiliations:** 0000 0001 2154 2448 grid.483500.a U. S. Food and Drug Administration, Center for Drug Evaluation and Research, Office of Translational Science, Office of Clinical Pharmacology, Division of Applied Regulatory Science, HFD-910, White Oak Federal Research Center, 10903 New Hampshire Ave, Silver Spring, MD 20993 USA

**Keywords:** mRNA, miRNA, Co-sequencing, Pancreatic injury, Biomarkers, Delayed anti-correlation

## Abstract

**Background:**

Co-sequencing of messenger ribonucleic acid (mRNA) and micro ribonucleic acid (miRNA) across a time series (1, 3, 6, 24, and 48 h post injury) was used to identify potential miRNA-gene interactions during pancreatic injury, associate serum and tissue levels of candidate miRNA biomarkers of pancreatic injury, and functionally link these candidate miRNA biomarkers to observed histopathology. RNAs were derived from pancreatic tissues obtained in experiments characterizing the serum levels of candidate miRNA biomarkers in response to acute pancreatic injury in rats.

**Results:**

No correlation was discovered between tissue and serum levels of the miRNAs. A combination of differential gene expression, novel delayed anti-correlation analysis and experimental database interrogation was used to identify messenger RNAs and miRNAs that experienced significant expression change across the time series, that were negatively correlated, that were complementary in sequence, and that had experimentally supported relationships. This approach yielded a complex signaling network for future investigation and a link for the specific candidate miRNA biomarkers, miR-216a-5p and miR-217-5p, to cellular processes that were in fact the prominent histopathology observations in the same experimental samples. RNA quality bias by treatment was observed in the study samples and a statistical correction was applied. The relevance and impact of that correction on significant results is discussed.

**Conclusion:**

The described approach allowed extraction of miRNA function from genomic data and defined a mechanistic anchor for these miRNAs as biomarkers. Functional and mechanistic conclusions are supported by histopathology findings.

**Electronic supplementary material:**

The online version of this article (10.1186/s12864-018-4657-2) contains supplementary material, which is available to authorized users.

## Background

MicroRNAs (miRNAs) are short ribonucleic acid (RNA) sequences that are hypothesized to primarily function as negative regulators of gene expression [1]. They accomplish this regulation largely through suppressing translation or catalyzing degradation of messenger RNAs (mRNAs) that serve as the template for protein synthesis in the cell [2]. Observed increases in serum miRNAs during multiple disease, dysfunction, and toxicity scenarios have stimulated interest in their use as non-invasive biomarkers of injury. The highly conserved nature of miRNAs across species and, therefore, their large translational potential have further heightened the scrutiny on these small RNAs especially those that are highly enriched in specific tissues [3]. Recent studies have demonstrated the potential of several pancreas-enriched miRNAs as non-invasive biomarkers of acute tissue injury with promise of high sensitivity and specificity [4–9]. Tools are constantly evolving to identify and provide validated interaction information for miRNAs and the mRNAs that they regulate [10]. Within these relationships lay the foundations for the hypothesis and design of future mechanistic investigations.

Next Generation Sequencing (NGS) provides an extremely powerful tool for generating comprehensive datasets for discovery and characterization of these interactive relationships [11, 12]. As the capability evolves to better analyze and interpret these NGS datasets, so does the ability to more completely capture all of the signaling, process, and regulatory elements in a given biological location at a specific point in time. Faced with these vast and complex NGS datasets, the scientific community, much as it did with microarray gene expression data, must refine quality parameters for, as well as the utility and shortcomings of, these datasets [13–16].

Previously, the caerulein model of acute pancreatic injury was used in rats and the magnitude and temporal responses of miR-216a-5p and miR-217-5p were characterized and their relationship to histopathology defined changes was reported [6]. These two miRNAs were evaluated because they are highly enriched in the pancreas. Therefore, any significant changes in their circulating levels would likely be due to pancreas specific injury and would represent a non-invasive and highly tissue specific marker of cellular injury. The present study was designed to interrogate paired NGS-generated miRNA and mRNA data across the experimental time points in this previous study with three objectives. The first objective was to identify the data defined signaling network, relevant canonical pathways, and potential miRNA-mRNA interactions during early, mild pancreatic injury. The second objective was to characterize the relationship of serum levels of specific candidate miRNA biomarkers of pancreatic injury, miR-216a-5p and miR-217-5p, to their tissue levels during pancreatic injury and recovery. The final objective was to interrogate the refined dataset and determine the miRNA-mRNA relationships of these two candidate miRNA biomarkers and compare the theoretical outcome of regulation of gene expression by these miRNAs to actual histopathology observations in the pancreas.

To achieve the present objectives, total RNA was exacted from frozen pancreatic samples generated in that previous study [6]. Extracted RNA was sequenced in separate runs for large and small RNA species to capture mRNA and miRNA results, respectively. This paired NGS data was analyzed for temporal change in expression patterns in both miRNA and mRNA over the first 48 h following caerulein induced pancreatic injury. The subset of miRNA that demonstrated significant expression change following treatment was examined for negative correlations in expression change with their predicted mRNA targets using a novel delayed anti-correlation approach. To satisfy the first study objective, pathway analysis was completed for the gene targets of all negatively correlated miRNA-mRNA pairs to identify potential components of a large miRNA-mRNA regulatory network in early pancreatic injury. The second objective was addressed by correlating quantitative real time polymerase chain reaction (qRT-PCR) measured changes of miR-216a-5p and miR-217-5p in serum with their NGS measured expression change in tissue across the same time points. The final objective was accomplished by demonstrating negative correlation between expression changes in miR-216a and miR-217 and expression changes in their predicted gene (mRNA) targets and then supporting these negative associations through the experimental literature. Subsequent investigation identified the functional pathways and processes associated with these literature-supported genes implicating these miRNAs in the regulation of those pathways and processes.

## Results

**Fig. 1:**
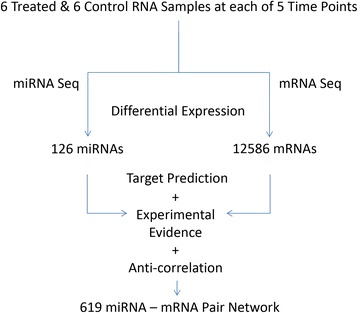
Research Scheme and Results of mRNA and miRNA Co-Sequencing during Pancreatic Injury. Co-sequencing revealed 126 miRNAs and 12,586 mRNA that were differentially regulated at some time in the experimental time course. Differentially expressed miRNAs were then linked to their predicted targets. Those predicted miRNA-mRNA pairs that had an anti-correlation formed a 619-pair signaling network active during pancreatic injury

**Fig. 2:**
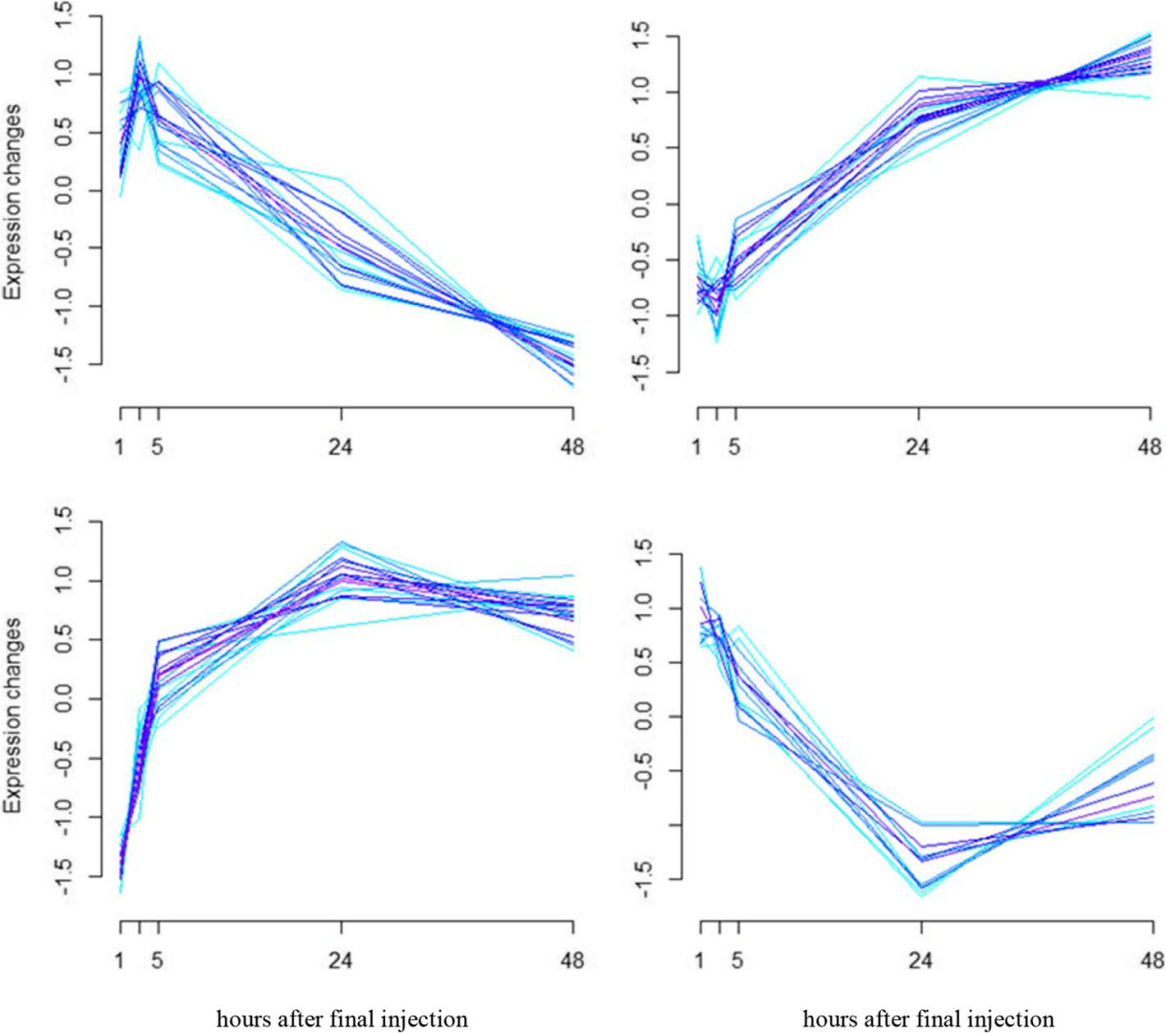
Temporal patterns of differential miRNA expression during pancreatic injury. Differentially expressed miRNA during pancreatic injury sorted into these 4 temporal patterns

**Fig. 3:**
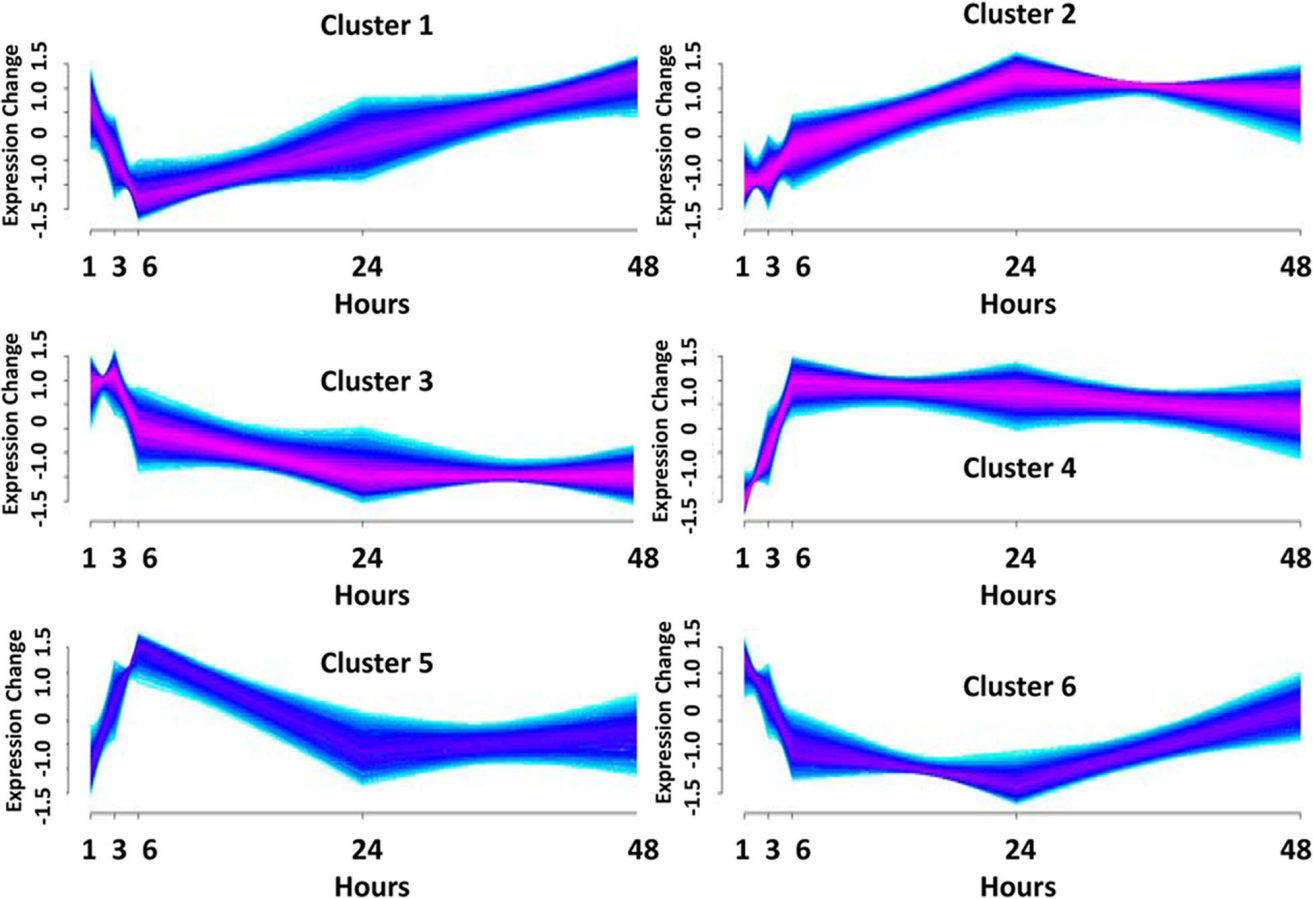
Temporal patterns of differential mRNA expression during pancreatic injury. Differentially expressed mRNA during pancreatic injury sorted into these 6 temporal patterns

Details of all the animal study methodology that yielded the samples from which the present molecular study were derived and the other findings from the animal study including all histopathology findings, characterization of miRNA levels in serum following acute pancreatic injury, and the relationship of the miRNAs to histopathology, have been previously published [6]. Figure 1 diagrams the analytical workflow and provides summary data for the present study on the scope of miRNA, mRNA, and potential miRNA-mRNA interactions defined in the data set. NGS identified 126 miRNAs and 12,586 mRNAs that were differentially expressed between treated and control samples in at least one time point (*p* ≤ 0.05). These data are supplied as additional materials (Additional files 1 and 2). Time series analysis of these differentially expressed RNAs revealed 4 distinct miRNA profiles (Fig. 2) and 6 distinct mRNA profiles (Fig. 3) during pancreatic injury and recovery. An integrated analysis combining computational miRNA target prediction, experimental miRNA target database interrogation, and miRNA/mRNA delayed anti-correlation of time profiles identified 619 miRNA-mRNA pairs (Additional file 3) that formed a regulatory network of miRNA-mRNA relationships with most miRNAs impacting multiple mRNAs. Pathway analysis suggested this network would affect cell survival/death-related pathways (e.g., Fibroblast Growth Factor and Epidermal Growth Factor mediated pathways) and immune response pathways (e.g., IL-2 and IL-6 signaling pathways). The top pathways associated with the network are listed in Table 1.

**Table 1:** Top pathways identified in large miRNA-mRNA defined signaling network

Pathway	Source	% of genes	False discovery rate
ErbB1 signaling	NCI	1.0	1.2e-6
Signaling by NGF	Reactome	3.8	7.2e-6
PIP3/AKT signaling	Reactome	0.9	7.2e-6
Signaling by EGFR	KEGG	0.7	6.2e-7
Signaling by SCF-KIT	Reactome	2.9	2.7e-5
IL-2 signaling	Reactome	2.6	2.7e-5
Signaling by PDGF	NCI	0.5	2.7e-5
Signaling by ERBB4	Reactome	3.0	2.7e-5
Signaling by FGFR3	Reactome	2.6	2.7e-5
IL-6 signaling	NCI	0.5	2.9e-5

**Fig. 4:**
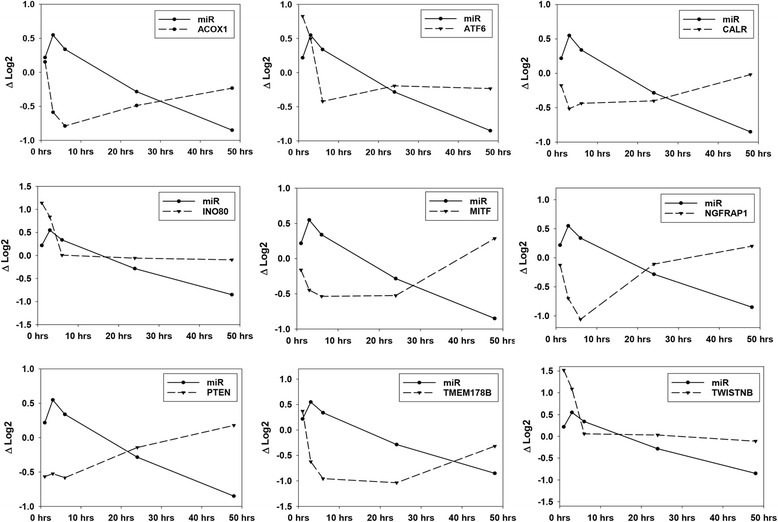
Genes of Differentially Expressed mRNAs Negatively Correlated to miR-216a-5p during Pancreatic Injury

**Fig. 5:**
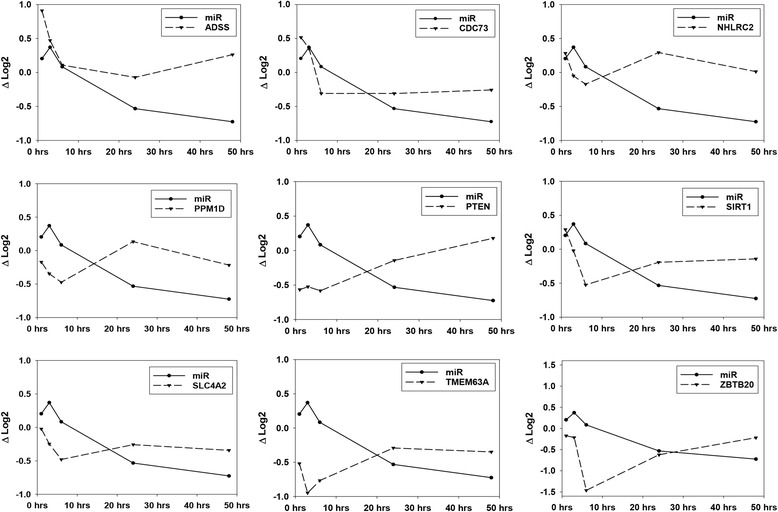
Genes of Differentially Expressed mRNAs Negatively Correlated to miR-217-5p during Pancreatic Injury

**Fig. 6:**
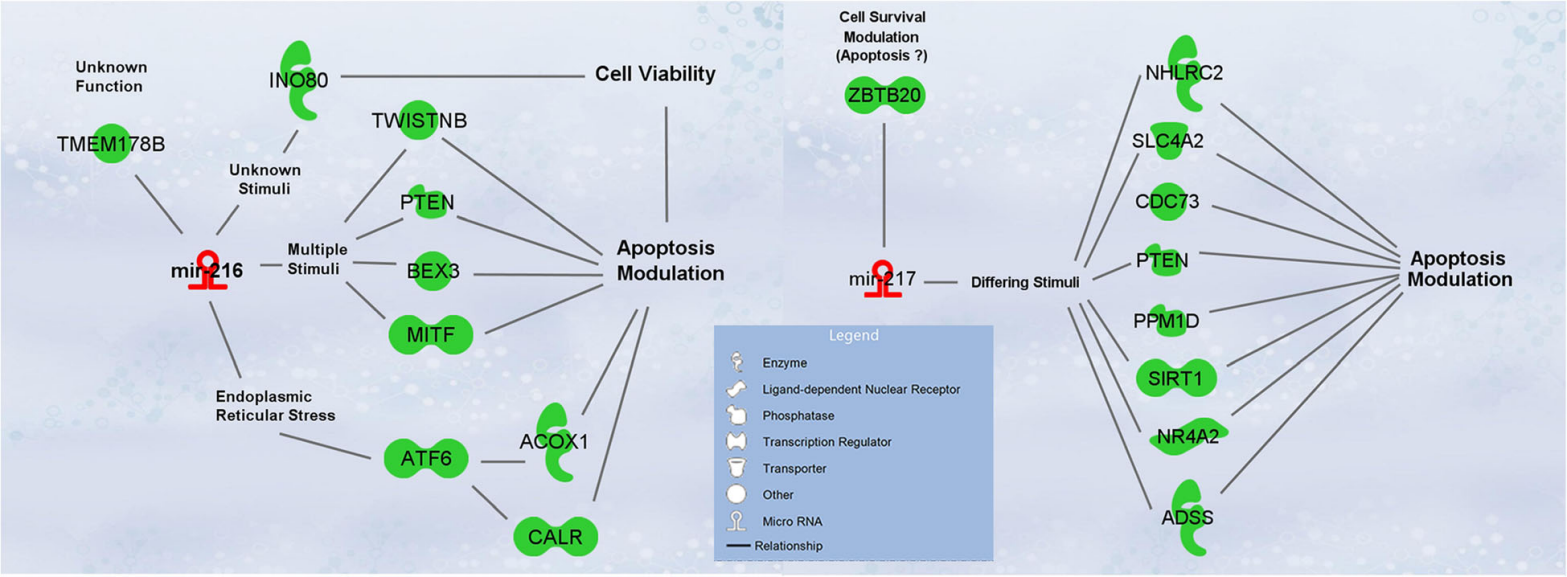
miR-216a-5p and miR-217-5p Associated Genes Modulate Apoptosis and Cell Survival. This figure shows previously reported relationships between mRNAs associated with miR-216a-5p and miR-217-5p in this study and modulation of apoptosis and cell survival, the key histopathology findings in the current study. Figure created with the pathway designer tool in IPA (QIAGEN Inc., https://www.qiagenbioinformatics.com/products/ingenuity-pathway-analysis)

Serum and tissue levels of two candidate miRNA biomarkers of acute pancreatic injury, miR-216a-5p and miR-217-5p, were compared across the time course (Additional file 4). Although serum levels of miR-216a-5p and miR-217-5p increased dramatically, no significant corresponding changes in tissue levels were detected at early time points. At 24 to 48 h post-treatment, as serum levels returned to or neared baseline levels, significant decreases in tissue levels were observed. No significant correlation was demonstrated between an animal’s tissue and serum levels of these miRNAs (Table 2). The relationships of these miRNAs to their presumptive mRNA targets were interrogated in this data set using a novel method that takes into consideration the possible delayed target gene expression change in response to its miRNA regulator (see Methods for details). This analysis reveals 9 mRNA negatively correlated with miR-216a-5p and 9 mRNA negatively correlated with miR-217-5p. These negative correlations are temporally depicted for miR-216a-5p and miR-217-5p in Figs. 4 and 5, respectively. The delayed anti-correlation scores (see Methods) for the individual miRNA-mRNA relationships are presented in Table 3. In Fig. 2, miR-216a-5p and miR-217-5p would be found in the group depicted in the upper left in which miRNA values initially increase followed by a gradual decline to or below control levels. Their associated mRNAs are immediately depressed relative to controls or decline after the miRNAs increase and then increase again as the miRNAs decrease. Relative to Fig. 3, these mRNAs would fall in clusters 1, 3, or 6. A focused pathway analysis of the down regulated genes associated with miR-216a-5p and miR-217-5p revealed that one mRNA (Pten) was a shared target of the two miRNAs and that all but two (Twistnb and Tmem178b) of the identified genes had some previously documented or hypothesized participation in pathways associated with cell autophagy and/or cell death stimulated by cellular stress (Fig. 6). Table 4 provides summaries of and references [17–58] for these associations.

**Table 2:** Pearson correlation analysis of tissue and serum miRNA levels

	miR-216a	miR-217
Coefficient	0.0324	−0.0274
*p*-value	0.806	0.835
n	60	60

**Table 3:** Time-shifted anti-correlation coefficients for miRNAs and verified target mRNAs

	ACOX1	ATF6	CALR	INO80	MITF	NGFRAP1	PTEN	TMEM178B	TWISTNB
miR-216a-5p	−0.9982	− 0.7833	− 0.8367	−0.9143	− 0.8788	−0.8744	− 0.7810	−0.9339	− 0.8513
	ADSS	CDC73	NHLRC2	PPM1D	PTEN	SIRT1	SLC4A2	TMEM63A	ZBTB20
miR-217-5p	−0.9368	−0.9337	−0.8931	− 0.7570	−0.7896	− 0.7178	−0.8062	− 0.9898	−0.8066

**Table 4:** miR-216a and miR-217 modulated mRNAs

Gene	Targeted by	Protein function
Atf6	miR-216a-5p	ER stress response transcription factor involved in autophagy and increasing chaperone proteins for unfolded proteins to enhance cell survival [18–22]
Ino80	miR-216a-5p	Component of chromatin remodeling complex; loss of activity yields decreased transcription of genes requiring this remodeling [23, 24]
Tmem178b	miR-216a-5p	Unknown function transmembrane glycoprotein
Acox1	miR-216a-5p	Beta-oxidation enzyme for long-chain fatty acids that are involved in ER stress responses resulting in apoptosis from mitochondrial injury & increased ROS [25–28]
CALR	miR-216a-5p	Calcium binding molecule that acts as a chaperone during endoplasmic reticulum stress and modulates apoptosis [29, 30]
Ngfrap1	miR-216a-5p	Mediator or co-factor inducing or promoting apoptosis in response to multiple signals [31, 32]
Mitf	miR-216a-5p	Transcription factor that when inhibited leads to apoptosis of mast cells and melanocytes; over expression can result in cellular hypertrophy [33, 34]
Twistnb	miR-216a-5p	Unknown specific function; component of RNA polymerase I (subunit RPA43) controlling transcription [35, 36]
Pten	miR-216a-5p miR-217-5p	A phosphatase mediator of apoptosis in multiple cell types and initiated through multiple signaling paths; loss of Pten leads to proliferation [37–40]
Zbtb20	miR-217-5p	Transcription factor associated with enhanced cell survival and proliferation [41, 42]
Sirt1	miR-217-5p	Enhances cell survival under stress and protects against apoptosis by promoting autophagy [43–46]
Slc4a2	miR-217-5p	Ion exchange protein that mediates anion influx into cells with loss of function promoting apoptosis [47, 48]
Nr4a2	miR-217-5p	Nuclear hormone receptor that modulates apoptosis with decreased expression yielding increased apoptosis [49, 50]
Cdc73	miR-217-5p	Component of transcriptional regulatory complex modulating apoptosis with loss of function and over expression associated with proliferation [51–53]
Nhlrc2	miR-217-5p	Asparagine-Histidine-Leucine repeat containing protein of undescribed function although this repeat motif is associated with caspase inhibitors and regulation of growth factors [54, 55]
Adss	miR-217-5p	Enzyme involved in purine synthesis and creation of AMP; reported in human autophagy network as a likely binding partner to ATG10, gene for an autophagy related enzyme [56, 57]
Ppm1d	miR-217-5p	Protein phosphatase implicated in regulation of apoptosis in pancreatic cancer and autophagy in genotoxic stress [58, 59]

*ER* endoplasmic reticulum, *ROS* reactive oxygen species, *AMP* adenosine monophosphate

**Fig. 7:**
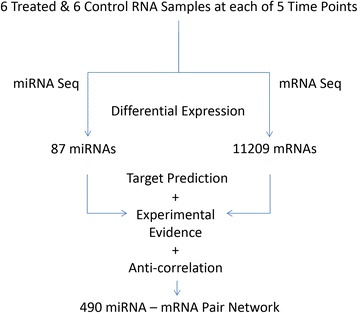
Impact of RNA Quality Bias on Results of mRNA and miRNA Co-Sequencing during Pancreatic Injury. This figure reflects fewer differentially expressed miRNAs and mRNAs and consequently fewer miRNA-mRNA pairs (see Fig. 1) when data were corrected for RNA quality bias

In processing samples, a correlation was suspected between sample RNA quality and gene counts from that sample as demonstrated in a RNA Integrity Number (RIN) plot across the time course (Additional file 1). RIN values were analyzed via two-way analysis of variance (ANOVA) revealing a strong interaction between time and treatment (*p* = 0.002) that obscured the relative contributions of each. Consequently, two tailed t-tests were run for each time point demonstrating that significantly higher RIN scores were observed in caerulein treated rats at 24 and 48 h post-treatment with *p*-values of 0.024 and 0.013, respectively. Since it has been reported that RNA integrity has a profound effect on measurements of gene expression levels [17], we repeated the Generalized Linear Model (GLM) analysis (see Methods) adding a RIN for each sample as a covariate in the model to control for the varying degree of RNA degradation among the samples and resulting in a change in the correlation of miRNA counts to RIN as shown in a bar graph (Additional file 2). This RNA quality bias correction modified the differential expression list generated by the model. This “corrected” differential expression list was then analyzed exactly as the original differential expression list and the differences between the two differential expression lists and their corresponding mRNA-miRNA relationships were examined. Figure 7 summarizes the workflow and findings following statistical correction for RNA quality bias. Compared with the uncorrected data in Fig. 1, the number of miRNAs and mRNAs identified as differentially expressed were decreased to 87 and 11,209, respectively (Additional files 5 & 6). The number of miRNA-mRNA pairs identified was reduced to 490 (Additional file 7). Following correction, the temporal relationships described in Figs. 2 and 3 had slightly reduced miRNA and mRNA numbers, but the shapes of the persisting relationships remained unchanged. RNA quality bias correction impacted the statistical analysis of miR-216a-5p and miR-217-5p so that significant expression change was not achieved although a significant expression change persisted for all but one of the target mRNAs; the expression of nhlrc2 was no longer significantly changed over any time points.

## Discussion

The linking of miRNA and mRNA sequencing to identify mechanism has become an increasingly popular tool in different fields of study in both *in vitro* and *in vivo* models [60–63]. Although experimental repeats may have been completed to demonstrate reproducibility, the majority of these studies focused on miRNA and mRNA differences in treatment groups at single time points or within single time point comparisons. These single time points have been logically chosen to provide the largest opportunity to capture relevant data but do not attempt to identify temporal relationships. Those studies that examine time series data [64, 65] rely upon intra-time point comparisons with the advantage of assessing changes for individual molecules from time point to time point but do not address how a change in one entity at one time point might impact a different entity at a different time point. The present study includes very acute time points, 1 h, 3 h, and 6 h, as well as time points 24 and 48 h post-treatment. Intra-time point comparisons in an acute time frame may not capture biological responses between miRNAs their mRNA targets that require some time for signaling, translation, and transcription upregulation. Therefore, the authors have created and applied a “delayed” correlation method that allows correlation assessment not only within the same time point but also across acute serial time points. This method will detect relationships that would not be evident within a single time point comparison. In our focused analysis of the data, a delayed response was often observed from the target gene after the change of the regulatory miRNA expression level.

However, the authors also recognize that even delayed anti-correlation analysis across 5 time points would not reliably identify miRNA-mRNA regulatory pairs or modules although others have published time series relationships with even fewer time points [11, 12]. Many random or “accidental” relationships would emerge. Therefore a standalone anti-correlation approach was not used. Instead, anti-correlation was only a component of a more comprehensive bioinformatics pipeline, as described in Methods, used to derive high-confidence miRNA-mRNA pairs. This pipeline included computational miRNA target analysis and experimentally verified target database interrogation to minimize or eliminate false positive identification of miRNA-mRNA pairs. At the end of the pipeline, the novel delayed method to evaluate miRNA/mRNA anti-correlation relationships was applied to identify miRNA/mRNA target pairs that are specifically linked to the biological process of interest in this study. The short list of target genes for each miRNA identified by this stringent bioinformatics pipeline is of high quality and biological significance, as evidenced by the fact the vast majority (15 out of 17) of identified targets for miR-216a-5p and miR-217-5p turn out to be associated with tightly regulated pathways related to injury response. The authors believe that this approach resulted in a list of high confidence pairs as demonstrated by the results for miR-216a-5p and miR-217-5p that were very strongly supported by histopathology findings. The integration of target prediction, time relationship profiles, and known functional relationships promoted the authors’ confidence in the data particularly in review of that the findings for miR-216a-5p and miR-217-5p where a connection could be made to the experimentally observed histopathology.

As hypothesized leakage serum biomarkers, the rapid increase in serum concentrations of the miRNAs with acute injury would be expected. The response of these miRNAs in injured tissue is less intuitive. In this study, no significant increase in tissue levels was noted at early time points. It may be that tissue levels are static or that the rapid (1 h or less) and progressive (over the first 6 h) leakage of miRNAs into the serum [6] might counter and obscure any concurrent increase in tissue levels at these early time points. Because these are proposed cell leakage biomarkers, it is likely that tissue expression levels will increase only if increase expression benefits the remaining intact cells. This is one reason that investigation of tissue levels is essential as it reflects regulation of cellular responses in a situation of stress whereas serum levels merely reflect what has leaked from an undetermined number of cells. That no clear relationship exists between tissue and serum levels neither supports nor contradicts the proposed value of these miRNAs as serum biomarkers of pancreatic injury. Significant tissue decreases in the miRNAs at later time points were recorded and seem consistent with a net loss of miRNAs to the serum, however, this was in no way demonstrated in this study. Further, at these later time points serum miRNAs levels had returned to baseline so ongoing leakage would not account for the decline and suggests reduced expression during recovery.

Initially, the total number (> 12,000) of mRNAs showing differential expression in this data set seems quite high. But it must be recognized that this represents differential expression at 5 time points across 48 h during which pancreatic injury evolution and resolution occurred as well as changes that would be expected with different times of the day and during different states of physiological activity. In that context, the total degree of differential expression (≈60%) does not seem unreasonable. When examined unfiltered, the relationship of the differentially expressed miRNAs and their differentially expressed presumptive target mRNAs, the resultant network was extremely complex and appeared largely indecipherable. Nevertheless, pathway evaluation identified a number of basic and critical cellular functions that would be influenced by this signaling network. However, finding specific discrete interactions that might provide targets for therapeutics or describe candidate biomarkers within this tangled network remains a daunting discovery task requiring evolving bioinformatics approaches.

A more focused interrogation of the co-sequencing data revealed association of candidate miRNA biomarkers, miR-216a-5p and miR-217-5p, with specific mRNA of genes involved in cell survival or death largely through autophagy and apoptosis. Confirmation of changes in respective protein levels or of the relationship of miR-216a-5p and miR-217-5p to autophagy and apoptosis or cell survival and death was not in the scope of this project. However, autophagy and apoptosis were the prominent processes observed in the tissue from which these RNA samples were obtained [6]. The balance of these processes within the tissue changed with severity of injury and determined survival or death for individual cells. These data and the analytical approach used to generate that data provide strong circumstantial evidence for mediation of autophagy and apoptosis in the pancreas by these specific pancreas enriched miRNAs. Definitive confirmatory experiments are warranted. Confirmation of these findings would provide a definitive mechanistic anchor for the use of these miRNAs as biomarkers in pancreatic injury as well as a definite function for them in pancreatic cells.

A potential bias in the study results based on a difference in RNA quality between treated and control animals (supplemental data) must be recognized. Visualization of control and treated RNA RIN scores suggested a difference between treatment groups. This quality difference based on treatment was identified using t-tests at each time point and the authors instituted statistical adjustments to counter bias as suggested by Romero et al., 2014 who indicated that using degraded samples can provide valuable data as long as there are not differences in RNA quality associated with treatment groups. Unfortunately, in this case, RNA quality was associated with treatment and some correction seemed appropriate. However, given a paucity of data and experience regarding correction, whether the application of these measures was an appropriate action is uncertain. Any validation of the correction in the large network analysis is difficult. In the specific cases of miR-216a-5p and miR-217-5p, the alignment of NGS described genes/pathways with the observed histopathology implies that the correction method eliminated information of a critical relationship in acinar cell injury response. While inequity in RNA quality between treatments would intuitively suggest a bias in differential expression meriting correction (a step not taken in most literature reports), the practical results in this case would suggest that correction was too harsh and removed genes (and miRNAs) with subtle but biologically meaningful differential expression arguing against correction. If this debate is to be settled, a better understanding of the impact and the boundaries of RNA quality bias on gene expression data is required as is additional knowledge of the physiological basis for RNA quality differences between the treatment groups observed in this study. In the present study, RIN was identified as significantly higher in caerulein treated animals at 24 and 48 h post-treatment. Intuitively there seems to be a biological basis for this pattern. As a part of response to injury, the buffering capacity and inactivation of enzymes in the pancreas is likely to be enhanced and to protect against degradation with the evidence of this protection being more evident in harvested samples as reactions are increasingly quenched at later time points. Further study is required to test this hypothesis.

## Conclusions

In conclusion, this study employed NGS, standard false discovery < 0.05, statistical parameters for differential expression, a novel delayed correlation analysis method, sequence based target prediction, and literature verification to define potential miRNA-mRNA interactions reflecting acute pancreatic cellular injury, evolution and resolution of changes in tissue morphology, and cell outcome (survival or death). Two candidate miRNA biomarkers of pancreatic injury, miR-216a-5p and miR-217-5p, were, thus, strongly linked to mRNA for genes previously associated with autophagy and apoptosis. The morphological changes observed in tissues in this study were autophagy and apoptosis. This study provides a theoretical mechanistic anchor for these miRNAs as biomarkers of pancreatic cellular injury, describes their potential function in pancreatic cells, and demonstrates an enhanced approach to extract function from genetic time series data.

## Methods

### Experimental design

As previously reported in greater detail [6], sixty male Sprague Dawley rats were randomly sorted into two experimental groups (treated and control) of thirty each. The groups received three subcutaneous injections one hour apart of either 50 μg/kg concentration caerulein (treated) or an equal volume of Dulbecco’s Phosphate Buffer (control). Six rats from each treatment group were then sacrificed at each of 5 time points; 1, 3, 6, 24, and 48 h after the final injection. At sacrifice, blood and pancreas were collected from each rat. A portion of each pancreas was placed in RNAlater (Life Technologies, Carlsbad, CA) for RNA isolation and NGS investigation and a separate portion of each pancreas was placed in buffered formalin for histopathology evaluation. The histopathology findings along with details of the animal study methods were published earlier [6]. Subsequently, NGS data were generated from the retained RNAlater samples and a functional interpretation of that data is presented here. Blood was allowed to clot and serum was separated and retained for miRNA extraction and subsequent quantification via RT-qPCR. Serum RT-qPCR quantification and tissue NGS derived tissue quantification of the miRNAs were compared to assess the serum-tissue relationship.

### MicroRNA isolation, preparation, and sequencing

RNA extraction, purification, and quantification were conducted in laboratories of the Division of Applied Regulatory Science at the White Oak Federal Research Center in Silver Spring, MD. Pancreas tissue from 60 rats was resected and preserved in RNALater (Life Technologies, Grand Island, NY), and stored at − 80 C. Six random batches of tissue samples were thawed, and 2.5 mg of each sample were processed using miRNeasy Mini Kit cat # 217004 (Qiagen, Valencia, CA). For equal loading, 5–10 mg of tissue were homogenized in 700 uL of Qiazol, for 5 min at 50 hz in a TissueLyser LT (Qiagen), and then diluted in Qiazol to a final concentration of 3.57 mg/mL. Then, 2.5 mg of tissue in 700 uL of Qiazol were processed using the automated purification of miRNA on a Qiacube (Qiagen), with the following protocol, “Purification of total RNA, including small RNAs, from animal tissues & cells (aqueous phase), version 2 (April 2010)” standard protocol, as described in the miRNeasy Mini Handbook (1,073,008 07/2012). Samples were eluted in 2 × 30 uL aliquots to maximize yields. Mean yield per 2.5 mg of pancreas tissue was approximately 150–200 μg RNA, determined by Nanodrop spectrophotometer (Thermo Scientific, Wilmington, DE). RNA quality RIN values were assayed by a 2100 Bioanalyzer Instrument (Agilent Technologies, Santa Clara, CA), using an Agilent RNA 6000 Nano Kit (cat # 5067–1511).

The sequencing of both mRNA and miRNA samples and initial quality control bioinformatic analysis were performed by Quintiles, Inc. (Morrisville, NC). Briefly, total RNA samples were converted into indexed cDNA libraries using TruSeq Stranded Total RNA sample preparation kit and TruSeq Small RNA sample preparation kit (Illumina, San Diego, CA) respectively. The libraries were quantitated by qPCR, normalized to 2 nM each, and sequenced on an Illumina HiSeq platform. For mRNA libraries 2 × 50 bp Paired-End sequencing configurations were used, while for small RNA libraries 1 × 50 bp Single End sequencing configurations were used. Eight samples were indexed and pooled together to be run on each lane, which were then demultiplexed after sequencing.

### NGS data analysis

Raw sequencing data (sra@ncbi.nlm.nih.gov; accession number SRP095173) was returned to our laboratory after going through Quintiles internal QC steps. In our laboratory, the reads from mRNA samples were aligned to the recently published [66] rat genome (ENSEMBL assembly 5.0) and transcriptome (ENSEMBL release 79) using START software version 2.4 [67] and then quantified using RSEM version 1.2.14 [68]. The reads from small RNA samples were collapsed to unique sequences and then aligned to miRBase [69] with Bowtie version 0.12.9 [70]. Differential expression analysis for both mRNA and miRNA were done using edgeR [71]. The gene/miRNA counts from RSEM above were upper-quartile normalized and fitted by a GLM with the group (treatment and time points) effects and other confounding factors as covariates. mRNAs or miRNAs that were differentially expressed between treated and time-matched control samples at any one of the five time points with a false discovery rate (FDR)-value ≤ 0.05 based on the negative binomial model were retained as differentially expressed and subjected to further analysis.

To enhance confidence in relationships and the resultant conclusions, anti-correlation across time profiles was combined with computational target prediction and experimentally based literature evidence for a relationship. Similar approaches have been previously published [11, 12] using fewer time points than those described within the present work. For each mRNA or miRNA in the differential expression list, the fold changes of expression levels between the treated and time-matched control samples across all five time points were analyzed by the R (http://www.r-project.org) package Mfuzz (http://mfuzz.sysbiolab.eu) to identify clusters of distinct time profiles following injury presented in Figs. 2 and 3. Targetscan version 7.0 [72] was queried to compile a list of computationally predicted target genes for each miRNA. All predicted targets whether evolutionally conserved or not were initially considered as target candidates. The predicted targets were further filtered through miRTarBase, a database collecting experimentally verified miRNA target genes [10]. Finally, a novel method to identify anti-correlation between time-shifted profiles was developed to capture possible delayed target gene expression changes in response to regulatory miRNAs. As a first step, for each miRNA or mRNA, a time series of expression data using the log2-transformed fold change between treated and control samples across the 5 time points was established. A differencing step was then applied to all time series by calculating the difference between two consecutive data points, resulting in differenced time series each with 4 data points. The Pearson correlation coefficients were calculated between time-matched differenced time series data from a miRNA and its target (called time-matched differenced coefficients in this method), and those miRNA/target pairs that showed a correlation coefficient greater than 0 were removed to ensure the remaining data were enriched with true miRNA/target pairs. As a last step, the differenced mRNA time series data were shifted (postponed) in relation to the miRNA series, resulting in a shifted match between the 1st, 2nd, and 3rd data points in the differenced miRNA time series and the 2nd, 3rd, and 4th data points in the corresponding mRNA time series, respectively. Pearson correlation coefficients were calculated between these time-shifted time series. The anti-correlation score of any miRNA/mRNA pair was defined as the minimum of the time-matched and time-shifted differenced coefficients. Any miRNA/mRNA pairs with an anti-correlation score less than − 0.7 were reported as the final regulatory miRNA/target pairs. Pathway enrichment analysis was performed using ReactomeFIViz (http://wiki.reactome.org/index.php/ReactomeFIViz), a Cytoscape (http://www.cytoscape.org) plugin for the Reactome Functional Interaction Database.

## Additional files

Additional file 1: Figure S1. This figure shows the quality of extracted RNA as measured by RIN for the control and caerulein treated animals throughout the experimental time course. ANOVA (p < 0.05) revealed that higher quality RNA was retrieved from control rats at 6 hours and from caerulein treated rats at 48 hours after treatment; RIN = RNA Integrity Number (a measure of RNA quality); Pt =Point (hours); Trx = caerulein treated. (PDF 61 kb)

Additional file 2: Figure 2. Correlation between RNA quality and miRNA(gene) counts. For each miRNA, the correlation coefficient between the vector of read counts and RINs across all samples was calculated. The distribution of these coefficients were plotted. Before removing RIN bias (Left), there are a number of miRNAs whose read counts are positively correlated with the sample qualities (coefficient > 0.5). After removing RIN bias (Right), all miRNAs have a count‐RIN coefficients < 0.5, suggesting the abundance of miRNAs is no longer correlated with RNA qualities. Num = number; RIN = RNA Integrity Number (a measure of RNA quality). (PDF 48 kb)

Additional file 3: mRNA-miRNA Anti-Correlation Values. List of values describing anti-correlation between mRNA and miRNA pairs. (TXT 24 kb)

Additional file 4: Table of Raw Values for Serum and Tissue Levels of MicroRNA Biomarkers. Table of individual animal values for microRNA biomarkers in the serum and in pancreatic tissue that demonstrated no serum to tissue correlation. (TXT 3 kb)

Additional file 5: Differentially Expressed miRNAs Following Correction for RIN Bias. List of miRNAs that were differentially expressed between treated and controls during at least one time point during the experimental time course following application of a statistical correction for treatment related difference in RNA quality. (TXT 74 kb)

Additional file 6: Differentially Expressed mRNAs Following Correction for RIN Bias. List of mRNAs that were differentially expressed between treated and controls during at least one time point during the experimental time course following application of a statistical correction for treatment related difference in RNA quality. (TXT 4114 kb)

Additional file 7: mRNA-miRNA Anti-Correlation Values Following Correction for RIN Bias. List of values describing anti-correlation between mRNA and miRNA pairs following application of a statistical correction for treatment related difference in RNA quality. (TXT 19 kb)

## Abbreviations

ANOVA: Analysis of variance
FDR: False discovery rate
GLM: General linear model
miRNA: microRNA
mRNA: messenger RNA
NGS: Next generation sequencing
qRT-PCR: quantitative real time polymerase chain reaction
RIN: RNA integrity number
RNA: Ribonucleic acid
