# Supplementary material for: Co-sequencing and novel delayed anti-correlation identify function for pancreatic enriched microRNA biomarkers in a rat model of acute pancreatic injury
Source: BMC Genomics. 2018 Apr 27;19:297. doi: 10.1186/s12864-018-4657-2 (PMC5922017; doi:10.1186/s12864-018-4657-2)
Supplement: Supplementary file 1 — This figure shows the quality of extracted RNA as measured by RIN for the control and caerulein treated animals throughout the experimental time course. ANOVA (p < 0.05) revealed that higher quality RNA was retrieved from control rats at 6 hours and from caerulein treated rats at 48 hours after treatment; RIN = RNA Integrity Number (a measure of RNA quality); Pt =Point (hours); Trx = caerulein treated. (PDF 61 kb) [file 12864_2018_4657_MOESM1_ESM.pdf]

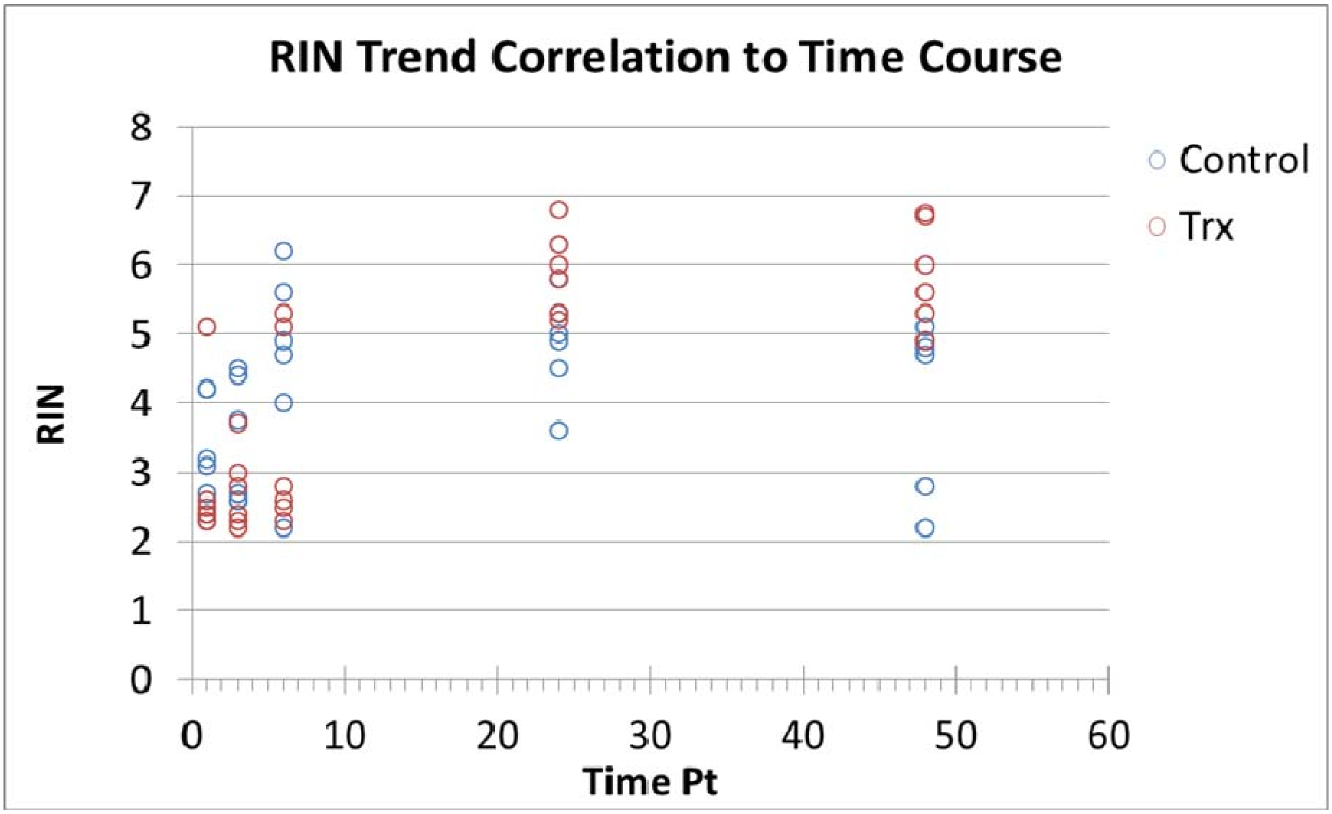

Fig S1. This figure shows the quality of extracted RNA as measured by RIN for the control and caerulein treated animals throughout the experimental time course. ANOVA ($p<0.05$) revealed that higher quality RNA was retrieved from control rats at 6 hours and from caerulein treated rats at 48 hours after treatment; RIN = RNA Integrity Number (a measure of RNA quality); Pt = Point (hours); Trx = caerulein treated.
