# Supplementary material for: Co-sequencing and novel delayed anti-correlation identify function for pancreatic enriched microRNA biomarkers in a rat model of acute pancreatic injury
Source: BMC Genomics. 2018 Apr 27;19:297. doi: 10.1186/s12864-018-4657-2 (PMC5922017; doi:10.1186/s12864-018-4657-2)
Supplement: Supplementary file 2 — Correlation between RNA quality and miRNA(gene) counts. For each miRNA, the correlation coefficient between the vector of read counts and RINs across all samples was calculated. The distribution of these coefficients were plotted. Before removing RIN bias (Left), there are a number of miRNAs whose read counts are positively correlated with the sample qualities (coefficient > 0.5). After removing RIN bias (Right), all miRNAs have a count‐RIN coefficients < 0.5, suggesting the abundance of miRNAs is no longer correlated with RNA qualities. Num = number; RIN = RNA Integrity Number (a measure of RNA quality). (PDF 48 kb) [file 12864_2018_4657_MOESM2_ESM.pdf]

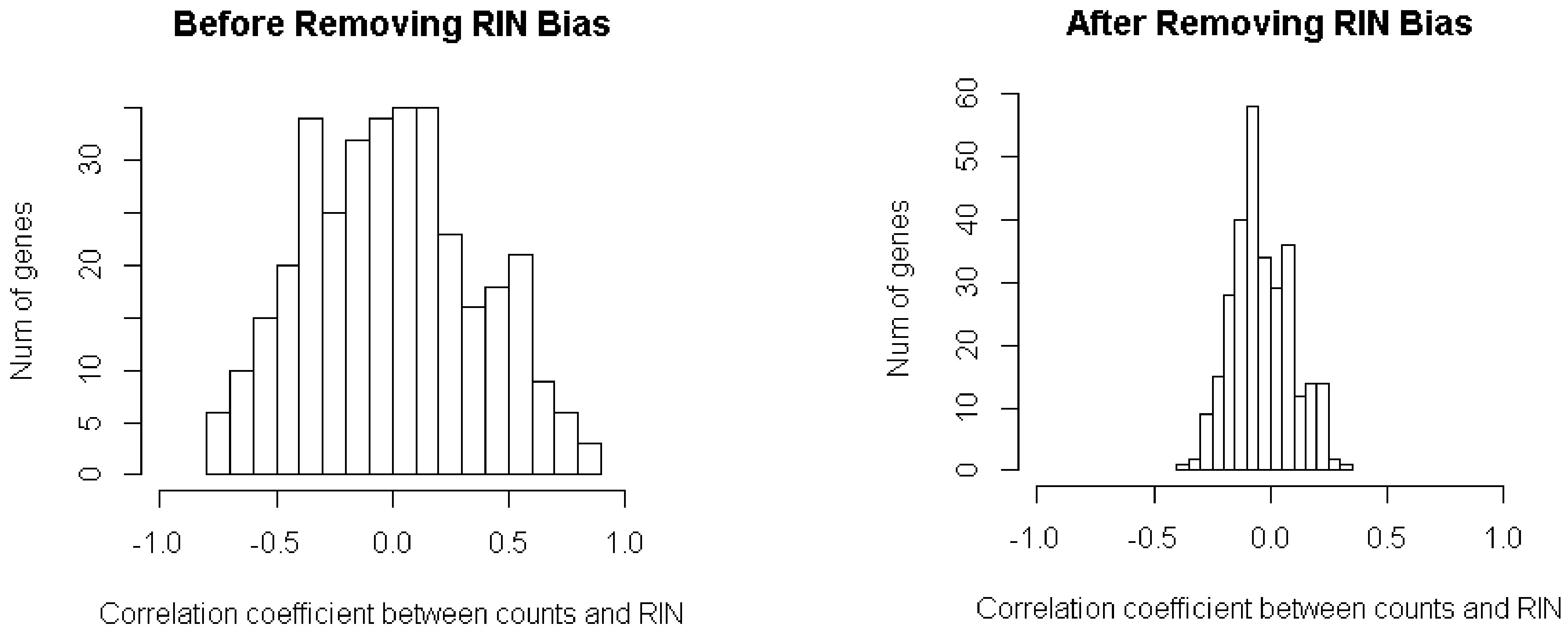

Additional Figure 2. Correlation between RNA quality and miRNA(gene) counts.

For each miRNA, the correlation coefficient between the vector of read counts and RINs across all samples was calculated. The distribution of these coefficients were plotted. Before removing RIN bias (Left), there are a number of miRNAs whose read counts are positively correlated with the sample qualities (coefficient > 0.5). After removing RIN bias (Right), all miRNAs have a count-RIN coefficients < 0.5, suggesting the abundance of miRNAs is no longer correlated with RNA qualities. Num = number; RIN = RNA Integrity Number (a measure of RNA quality)
